# A Case Report of Impending Thyroid Storm and Pemphigoid Gestationis Complicating Complete Molar Pregnancy

**DOI:** 10.7759/cureus.91790

**Published:** 2025-09-07

**Authors:** Yaw Abrefa Safo, Dorothy Sackey, Theophilus B Sabi

**Affiliations:** 1 Obstetrics and Gynaecology, Tema General Hospital, Tema, GHA; 2 Pediatrics, Battor Catholic Hospital, Battor, GHA; 3 Obstetrics and Gynaecology, Princess of Wales Hospital, Bridgend, GBR

**Keywords:** gestational trophoblastic disease, molar pregnancy, pemphigoid gestationis, skin disease, thyroid storm

## Abstract

Gestational trophoblastic disease, very rarely, is complicated by thyroid storm and pemphigoid gestationis. Thyroid storm is related to human chorionic gonadotropin hormone levels produced in pregnancy, but is usually exaggerated in molar pregnancies. With thyroid storm presenting acutely with cardiovascular and gastrointestinal collapse and pemphigoid gestationis manifesting as an intense, blistering, pruritic dermatosis, it is critical to recognise early the signs and symptoms to avert complications from these rare phenomena. We present a 24-year-old female who was referred with amenorrhoea of three months' duration with persistent vomiting, diarrhoea, and palpitations. She spontaneously expelled huge vesicular moles and subsequently developed pemphigoid gestationis. Skin biopsy showed focal spongiosis with subepidermal bullae and an admixture of eosinophils and neutrophils extending deep into the dermis with associated hyperkeratosis as well as intraepidermal blood-filled bullae. She was managed by a multidisciplinary team with prompt action to prevent deterioration.

## Introduction

Pregnancy is associated with various physiological changes, and this includes activation of thyroid-stimulating hormone receptors by human chorionic gonadotropin (hCG) hormone, as they are structurally similar. Hyperthyroidism occurs in about 0.2-0.7% of all pregnancies and about 25-65% of molar pregnancies, very rarely progressing to the life-threatening complication of thyroid storm [1]. Thyroid storm tends to occur when the patient’s ability to compensate for cardiovascular, metabolic, and thermoregulatory systems in the hyperthyroid state is exceeded [2]. Pemphigoid gestationis (PG) is a rare, self-limiting, autoimmune bullous dermatosis of pregnancy [3] often presenting with intense pruritus and an erythematous papular rash. It is usually caused by immunoglobulin G1 (IgG1) autoantibodies directed against collagen XVII, a glycoprotein within the basement membrane of the epidermis [4]. It has an incidence of 1:10,000 to 600,000 pregnancies [5]. Diagnosis of pemphigoid gestationis is mainly done by a combination of clinical history, examination, skin biopsy, and/or enzyme-linked immunosorbent assay [6].

Gestational trophoblastic disease has an incidence of 1:500 pregnancies [7]. Once confirmed, it requires dilatation and suction evacuation followed by histopathological examination of the contents evacuated and regular monitoring for resolution of both symptomatic and biochemical findings.

## Case presentation

A 24-year-old female, nulliparous, was rushed into the gynaecology emergency unit, Tema General Hospital (TGH), Tema, with amenorrhoea of three months' duration. She had vomited twice that morning prior to the presentation. She also had mild abdominal pain, crampy in nature, non-radiating, with no aggravating or relieving factors, and rated as 3/10 on the pain score scale. This was associated with fever, occasional sweating, diarrhoea, and palpitations. There was no bleeding per vaginum. She had a history of one spontaneous miscarriage a year prior to presentation. She had no relevant past medical, surgical, or drug history, including intake of herbs. She had taken neither alcohol nor any illicit drug prior to the presentation.

On arrival, she was anxious, mildly pale, febrile, not jaundiced, and moderately dehydrated with pale, dry, scaly skin and no pedal swelling. She had a blood pressure of 157/107 mmHg, a regular pulse of 119-121 bpm, a saturation of 98% on room air, and a temperature of 39.80°C. No anterior neck mass was palpated, and she had no exophthalmos. First and second heart sounds were present and normal, and the respiratory rate was 26 cycles per minute with normal chest sounds. The abdominal examination showed a uterine size of 16 weeks, larger than the estimated gestational age of approximately 12 weeks and four days. The Glasgow Coma Score (GCS) was 15 out of 15.

A urine pregnancy test was positive, and samples for full blood count (FBC), venous blood gas, blood film for malaria parasites (BF for MPs), blood grouping and cross matching, quantitative serum beta human chorionic gonadotropin (B-hCG), thyroid function test (TFT), baseline renal and liver function test and blood culture were carried out (Table 1). The chest X-ray appeared normal with normal lung fields and cardiac silhouette, and the electrocardiogram (ECG) was also normal. No antecedent cause of the fever was found.

**Table 1:** Pattern of test results from day of admission to outpatient follow-up WBC: White Blood Cell, BhCG: Beta human Chorionic Gonadotropin, UREA: Urea, CR: Creatinine, AST: Aspartate Aminotransferase, ALT: Alanine Aminotransferase, TSH: Thyroid Stimulating Hormone

TEST RESULT	Normal range	DAY 1	DAY 2 (24HRS POST EXPULSION)	DAY 5	DAY 14 (day of discharge)	Week 8	Week 10
Hemoglobin(g/dl)	11.5-16	10.1	12.9	-	13.4	-	-
WBC (10^9^/L)	3-8	21	16	9	-	-	-
BhCG (IU/L)	0.5-5	109,831	66,083	-	3,573	<5	<5
UREA (mol/L)	2-7.5	11.58	9.3	5.1	-	-	-
CR (µmol/L)	53-97	171.04	101	93	-	-	-
AST (U/L)	5-34	48	29	-	-	-	-
ALT (U/L)	10-36	66	32	-	-	-	-
TSH (mIU/L)	0.5-5.0	<0.4	0.66	-	-	-	-
T3 (pmol/L)	1.0-2.8	>10	4.91	-	-	-	-
T4 (nmol/L)	58-140	220.25	72.28	-	-	-	-

**Figure 1:**
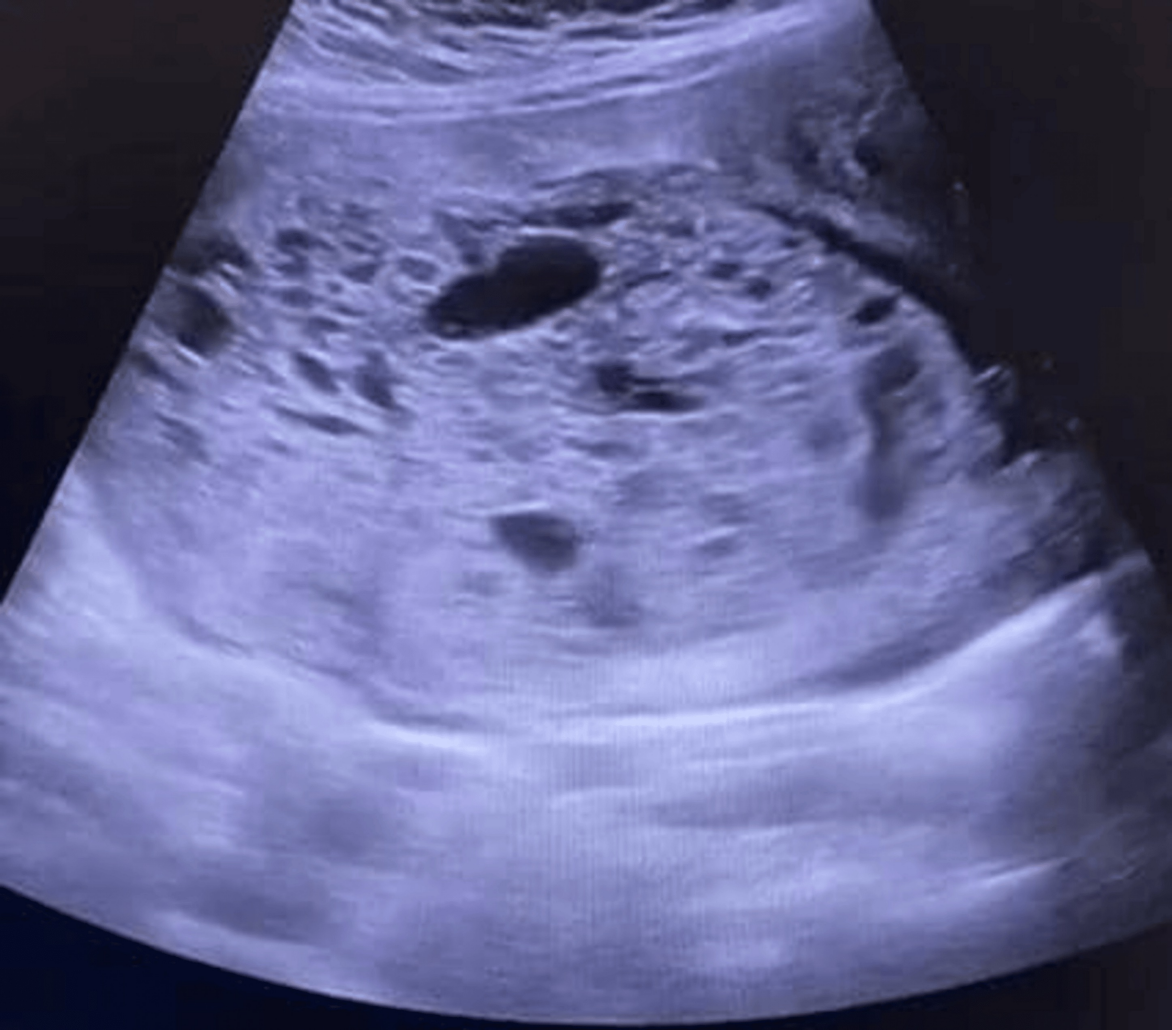
Ultrasound showing “cluster of grape-like” appearance

She was quickly admitted to the high-dependency unit (as our intensive care unit had no bed spaces) and stabilised with intravenous fluids and broad-spectrum antibiotics (Ceftriaxone 2g daily) while awaiting the blood tests. An abdomino-pelvic ultrasound (Figure 1) done showed normal abdominal findings but an enlarged uterus measuring 15.3x10.3x12.0 cm with no gestational sac or foetal pole, but rather an echogenic mass having multiple cystic appearances suggestive of a complete hydatidiform mole. Both ovaries appeared normal with free fluid in the pouch of Douglas.

With such a deranged thyroid function test, a Burch-Wartofsky score [8] was quickly calculated, and the patient scored 45, with temperature scoring 25, gastrointestinal hepatic dysfunction scoring 10, and heart rate scoring 10. A diagnosis of impending thyroid storm was therefore made. Long-acting nifedipine 30mg daily, propranolol 40mg twice daily, methimazole 20mg twice daily (as propylthiouracil was unavailable), and five drops of Lugol's iodine 2% every eight hours were given.

She was then assessed by an anaesthesiologist with endocrine input and subsequently counselled on the condition, risk factors, and complications, and the need for dilatation and suction evacuation to be carried out in theatre after stabilising her. Some hours into admission, the patient spontaneously expelled after suddenly complaining of worsening lower abdominal pain and bleeding. Bleeding continued, and she was taken to the theatre for gentle suction evacuation under sedation and ultrasound guidance to ensure complete evacuation. She was transfused with two units of packed cells intraoperatively. The histopathological report confirmed a diagnosis of complete hydatidiform mole after demonstrating the absence of foetal parts. She gradually improved in both clinical and biochemical parameters.

**Figure 2:**
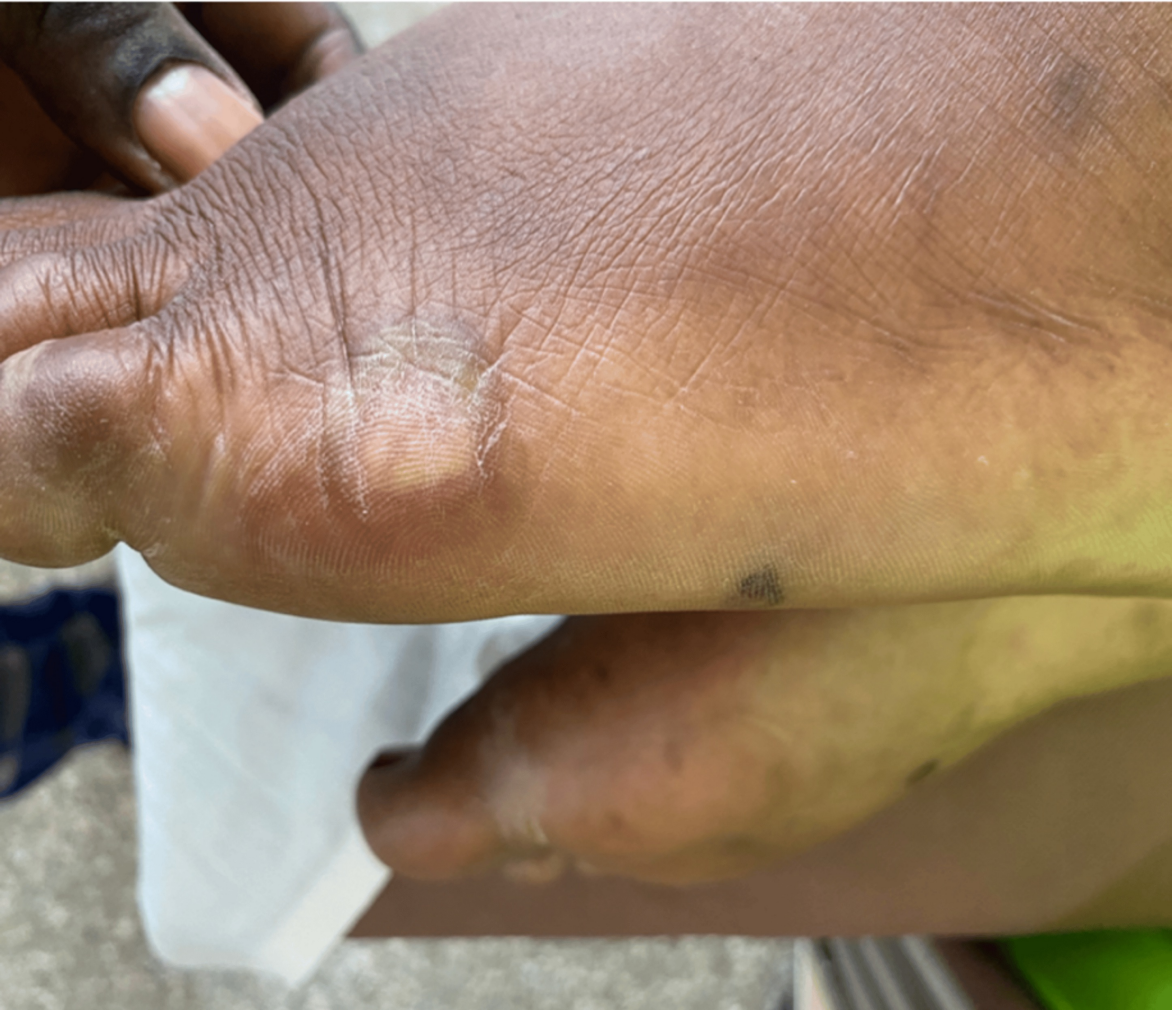
Skin lesion on lateral aspect of left foot

**Figure 3:**
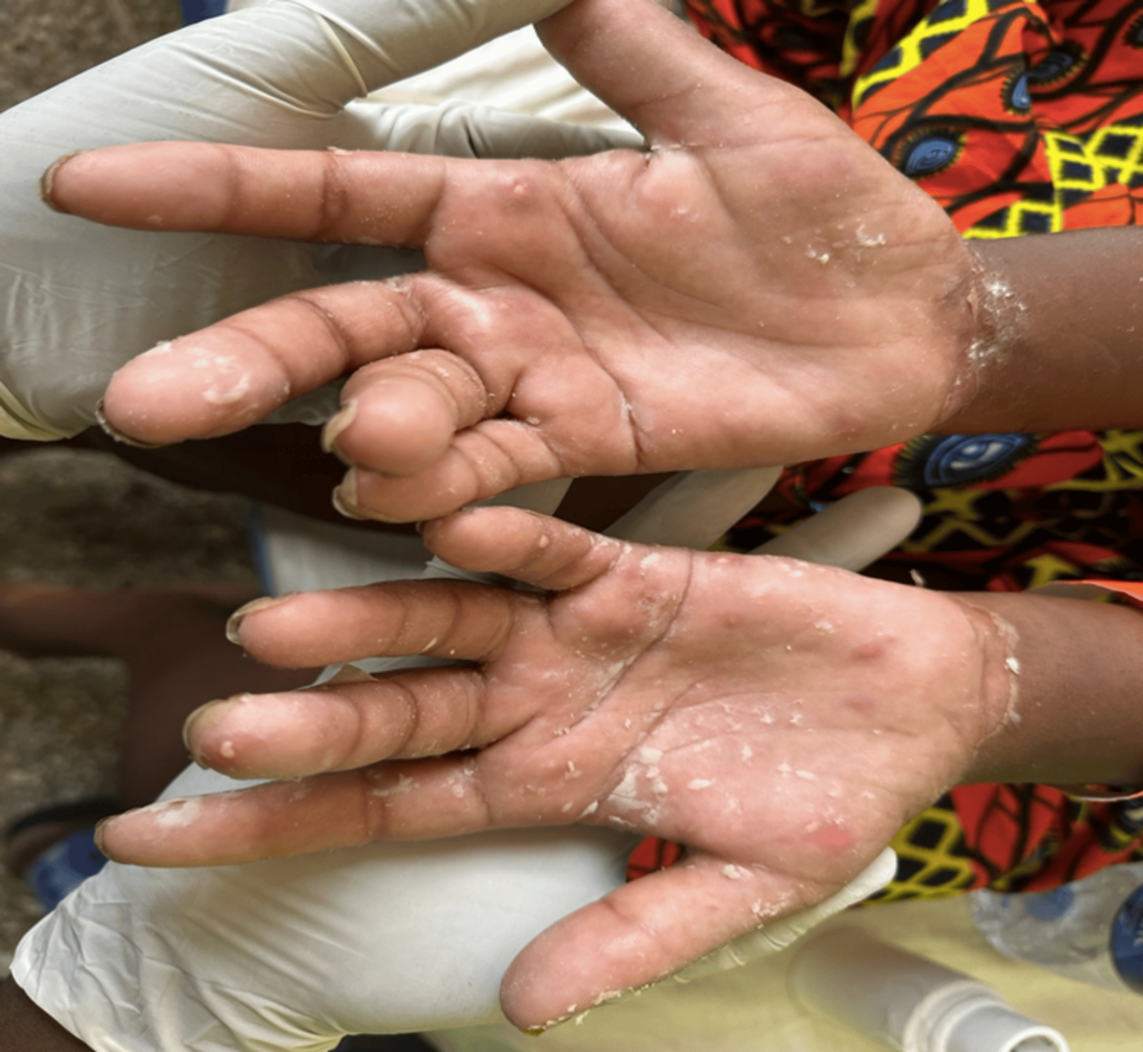
Bilateral erythematous palmar lesions

**Figure 4:**
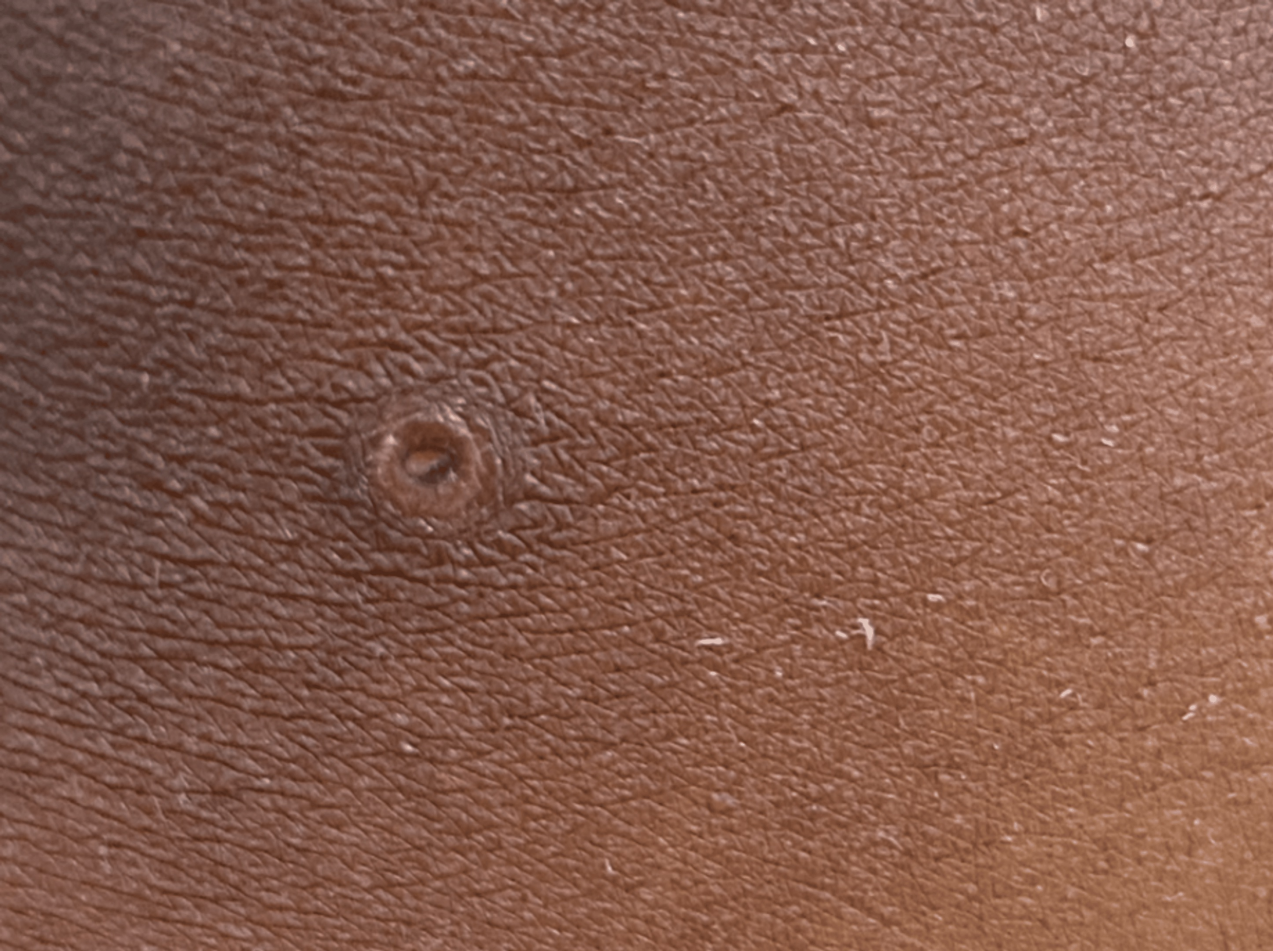
Ruptured vesicular lesion on skin

**Figure 5:**
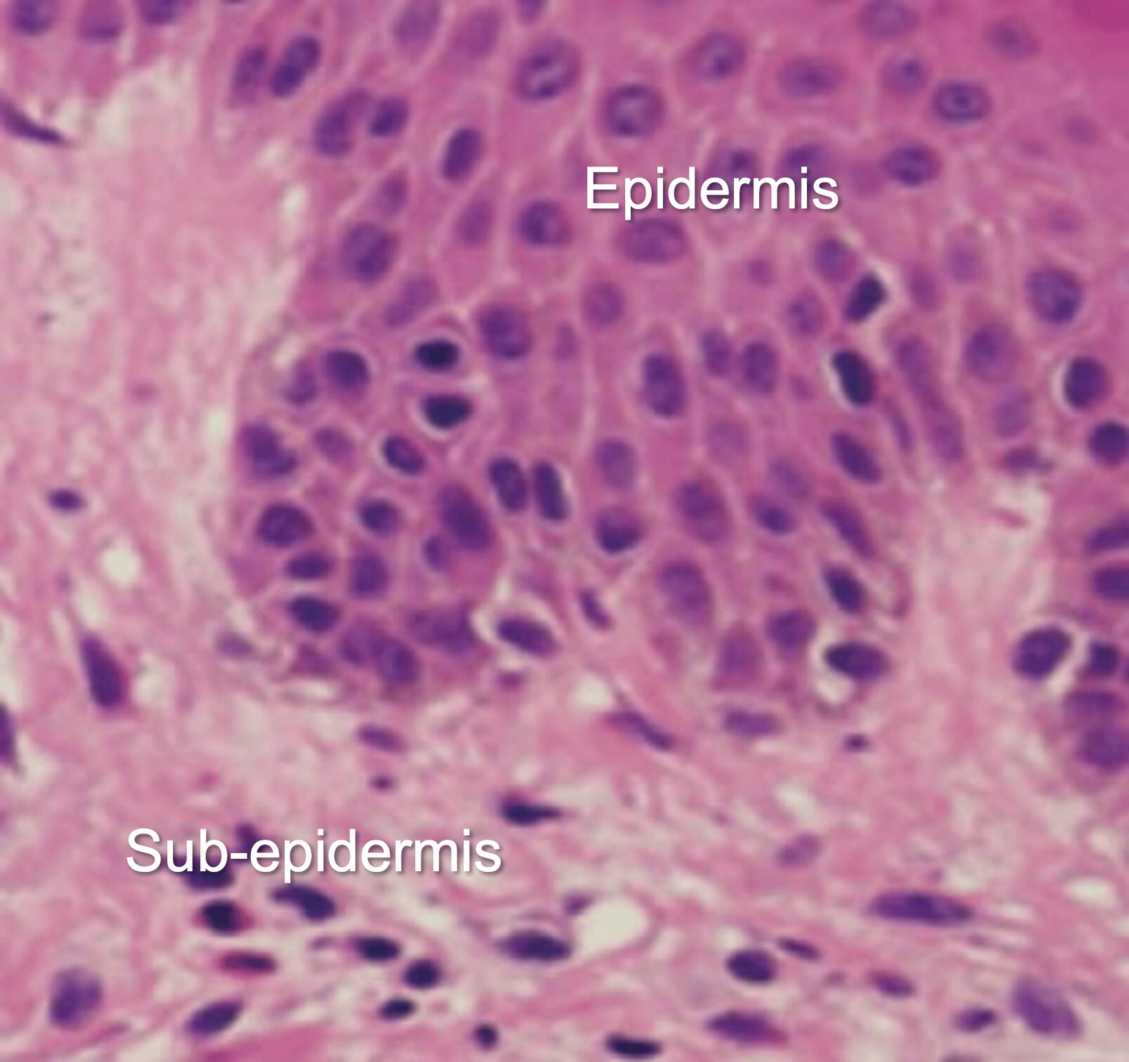
Histopathology image showing sub-epidermal bullae and admixture of eosinophils and neutrophils

Three days post-evacuation of the vesicular mole, she developed erythematous papular rashes on her body and the dorsal and palmar surfaces of her hands and feet (Figures 2-4). A skin biopsy was done after the skin lesions rapidly became worse, and this showed focal spongiosis with subepidermal bullae containing an admixture of neutrophils and eosinophils and extending deep into the dermis (Figure 5), where foci of suppuration are seen. There were intraepidermal blood-filled bullae in some areas. A perivascular inflammatory infiltrate was also noted with mild hyperkeratosis, and this was in keeping with pemphigoid gestationis. She was started on oral prednisolone 40 mg daily with resolution of skin lesions by the 14th day after admission, and she was subsequently discharged.

She was seen weekly with repeat serum B-hCG and thyroid function tests. The thyroid function test normalised by day two, and B-hCG reached undetectable levels by week eight after evacuation. It remained undetectable on two subsequent values.

## Discussion

In Ghana, data on the prevalence of gestational trophoblastic disease are scanty; however, in the United States, it occurs in approximately 121 per 100,000 pregnancies [9]. These patients are at risk of hyperthyroidism and its complication, thyroid storm, even though this is rare [10], occurring in 5% of cases of molar pregnancies [11]. Thyroid-stimulating hormone (TSH) and hCG are structurally similar in the alpha units, allowing hCG to bind to TSH alpha receptors and exert its effect on thyroid membranes [12]. In a molar pregnancy, the level of hCG is exaggerated and higher than that of a normal pregnancy state. This, therefore, increases the risk of hyperthyroidism and thyroid storm in molar pregnancy.

Diagnosis of impending thyroid storm is clinical, and investigations, though important, do not distinguish thyroid storm from impending thyroid storm, as in our case. An impending thyroid storm was diagnosed in our patient after being admitted to our high dependency unit (HDU), using Burch-Wartofsky criteria (BWPS).

Another criterion also available is the Japanese Thyroid Association; however, the BWPS takes into consideration the precipitant history and systemic effects of the hyperthyroidism and their compensatory status. These systems are the patient’s thermoregulatory dysfunction, central nervous system effects, gastrointestinal-hepatic dysfunction, and cardiovascular dysfunction [13]. Using the BWPS criteria, she scored 45, highly suggestive of impending thyroid storm, and using the Modified Early Obstetrics Warning Score (MEOWS), the severity of her condition and her status were monitored. She was triaged as red based on the score of 15.

Prompt emergency assessment of airway (A), breathing (B), circulation (C), and disability (D) is the first step, correcting any abnormalities as one moves along. Treatment is no different from the non-pregnant state, with the only difference being that, after stabilisation, evacuation of the uterus ultimately remains the definitive form of treatment that needs to be performed to ensure resolution of symptoms from the thyroid storm. Intravenous access is secured, and hydration is started, as most of these patients are very dehydrated. Therapy is usually continued with propylthiouracil (PTU) 400-600 mg stat, orally, followed by 150-200 mg every four to six hours. In our case, due to the unavailability of PTU, methimazole was used. PTU is better in cases of thyroid storm, as it also helps prevent peripheral conversion of free T4 (FT4) to free T3 (FT3) (IV dexamethasone 2 mg every six hours also works by a similar mechanism). Oral methimazole inhibits the production of new thyroid hormones from the thyroid gland. Methimazole inhibits the thyroid peroxidase enzyme, which is responsible for the organification of iodine and the coupling of thyroglobulin molecules. It is 10x more potent than propylthiouracil [14].

Propranolol 40 mg, a non-selective beta blocker, not only slows down the heart rate but also inhibits the peripheral conversion of FT4 to FT3. Propranolol’s peripheral conversion ability is achieved by inhibiting the enzyme 5’-monodeiodinase. This makes propranolol more effective than other beta blockers such as metoprolol [15]. Propranolol, with its beta-two blocking effect, also creates an increase in systemic vascular resistance, reducing circulatory collapse [14].

Iodide products further suppress FT3 and FT4 release from the thyroid gland. Options include sodium iodide, potassium iodide, and Lugol’s solution. Five drops of Lugol’s iodine were given in our case, inhibiting iodine organification. The administration of PTU/methimazole, at least an hour before iodide products, leads to avoidance of the Jod-Basedow effect, an abrupt rise in T4 formation and release, whilst inducing the Wolff-Chaikoff effect, a transient inhibition of iodine organification [8].

Three days post-evacuation, our patient developed a generalised dry erythematous maculopapular rash, but more prominent in her palms, feet, and chest. Some of these lesions are transformed into vesicles and bullae. A skin biopsy from three separate areas was taken, which confirmed the diagnosis of pemphigoid gestationis. Due to the financial constraints, serum immunoglobulin E and anti-BP 180-NC16a antibody index, which are usually high in PG, could not be performed. Hence, the diagnosis of PG was limited to only the histopathological diagnosis.

PG is a rare autoimmune blistering dermatosis initially named “herpes gestationis”, but now it is a misnomer, as it is not related to herpes infection. It is an autoimmune condition because autoantibodies are directed against hemidesmosomal protein BPAG2, also called collagen XVII, a 180-kD antigen located in the skin. This causes complement C3 to be directed and deposited at the basement membrane of the dermo-epidermal junction (DEJ). Along with C3, these autoantibodies and T-cells are also directed to the extracellular region of the bullous pemphigoid antigen 2 (BPAG2) near the membrane called the MCW-1 domain [16]. This region is also a key immunodominant epitope in bullous pemphigoid, a closely related autoimmune blistering disease.

In our patient, a differential diagnosis of polymorphic eruption of pregnancy (PEP) was considered; however, PG can be distinguished from it, as it starts around the periumbilical area and spreads, while PEP usually begins in the striae and spares the umbilicus. Again, the skin biopsy showed focal spongiosis with sub-epidermal bullae extending deep into the dermis, where foci of suppuration were seen. There were also intra-epidermal blood-filled bullae in these areas. A perivascular inflammatory infiltrate was also noted with mild hyperkeratosis; this was in keeping with pemphigoid gestationis. Steroids are usually the mainstay of management.

Our patient was started on prednisolone 40 mg daily, leading to resolution of the skin lesions and successful discharge, where she continued both dermatological, endocrine, and gynaecological reviews.

## Conclusions

An impending thyroid storm and pemphigoid gestationis are extremely rare complications of gestational trophoblastic disease. Impending thyroid storm can lead to life-threatening complications such as acute decompensated heart failure, respiratory failure, and thermoregulatory dysfunction, while pemphigoid gestationis, a distressing pruritic disease, could have a debilitating effect on patients’ skin and recovery. Hence, a prompt and urgent diagnosis, as well as close monitoring, is required to enhance recovery.
